# Cervical Human Papillomavirus Prevalence, Genotypes, and Associated Risk Factors among Female Sex Workers in Greater Accra, Ghana

**DOI:** 10.1155/2019/8062176

**Published:** 2019-06-02

**Authors:** Abdul Rashid Adams, Priscillia Awo Nortey, Benjamin Ansah Dortey, Richard Harry Asmah, Edwin Kwame Wiredu

**Affiliations:** ^1^Department of Medical Laboratory Sciences, School of Biomedical and Allied Health Sciences, College of Health Sciences, University of Ghana, Korle-Bu, Accra, Ghana; ^2^School of Public Health, College of Health Sciences, University of Ghana, Legon, Accra, Ghana; ^3^Trauma Hospital, Winneba, Ghana; ^4^Department of Pathology, School of Biomedical and Allied Health Sciences, College of Health Sciences, University of Ghana, Korle-Bu, Accra, Ghana

## Abstract

Cervical cancer is a largely preventable disease mediated by persistent infection with high-risk Human Papillomaviruses (Hr-HPV). There are now three approved vaccines against the most common HPV genotypes. In Ghana, mortality due to cervical cancer is on the rise, due to the absence of an organized and effective cervical cancer prevention and control program. Data on circulating HPV genotypes is important for studying the likely impact of mass introduction of HPV vaccination of the female population before sexual debut. High HPV prevalence has been reported in Female Sex Workers (FSWs), who constitute an important active group for maintenance of HPV in the population. This study was conducted to determine the size of HPV prevalence in this group and to provide information for future assessment of the impact of vaccine introduction in the country. We conducted a cross-sectional study where the snowballing technique was used to identify and select FSW's ≥18 years, operating within suburbs of Greater Accra Region (GAR). A risk factor assessment interview was conducted and cervical swabs were collected for HPV-DNA detection and genotyping by Nested Multiplex PCR. Hundred participants, age ranging from 18 to 45 years, median 24 years, were studied. The prevalence of Cervical HPV was 26%. Eleven genotypes were detected comprising 9 high-risk in order of decreasing prevalence HPV-16 (8%), HPV-35 (5%), HPV-33/39/-68 (3%), HPV-52/51/59 (2%) and HPV-18 (1%) and 2 Low-risk types, HPV-42(3%), and HPV-43 (1%). Three women had HPV types that could not be genotyped by our method. Oral contraceptives use was associated with a reduced chance of HPV infection (P=0.002; OR=0.19, 95% CI 0.07-0.54). This study found a high HPV prevalence among FSWs in the GAR. A high number of Hr-HPV genotypes seen are vaccine preventable, providing additional compelling argument for implementing a national cervical cancer prevention plan including vaccination.

## 1. Introduction

Globally, cervical cancer remains a primary cause of morbidity and mortality, with estimated 569,847 new cases and 311,365 attributable deaths in 2018 [1, 2]. The highest incidence has been reported in low and middle income countries, particularly in Sub-Saharan Africa where it is the second most common female malignancy [3]. In Ghana, it is likely the commonest cancer among women. Current estimates indicate that every year 3,151 women are diagnosed with cervical cancer and 2,119 die from the disease in the country [4].

Infection of the cervix by a high-risk Human Papillomavirus (HPV), a common sexually transmitted infection, is necessary for the development of cervical cancer [5]

Many studies have found a direct association of HPV infection with sexual behaviour and have indicated that a high number of lifetime partners may lead to a higher transmission of HPV leading to higher cervical cancer rates [6–8].

Female sex workers (FSWs) are a group of females who provide sexual services for economic remuneration. Due to exposure to multiple sexual partners in their occupation, they are prone to various sexually transmitted infections, including HPV. Sexual contact with FSWs plays an important role in HPV transmission and might be a major contributor to the prevalence of HPV and cervical cancer among women in the general population. Also, through the transmission of the virus to their male clients, they increase the risk of penile cancers among these men [9]. Furthermore, due to their likely interaction with foreign clients and sex tourists, they may possibly have a role in the genotype diversity of HPV in the country.

In Ghana, mortality due to cervical cancer is on the rise, most likely due to the lack of an organized prevention and control program. Information on circulating HPV genotypes is crucial for determining the impact of cervical cancer control programmes, including HPV immunization. The high rates of HPV reported among FSWs make them a priority group for study as we seek to characterize the prevalent HPV genotypes in the country in order to predict the likely impact of the current vaccines in reducing the incidence of cervical cancer after introduction of mass female vaccination in the country. The study also looked at other factors that may increase the risk of HPV, contribute to persistence of infection, and/or promote progression of HPV-induced changes in the cervical epithelium. Here, we report the first study of cervical HPV and its associated risk factors among FSW's in Ghana.

## 2. Methods

### 2.1. Study Design and Population

This was a cross-sectional study undertaken between February and July 2016 in Greater Accra, one of the ten administrative regions of Ghana (and which houses the national capital) with a predominantly urban population. A risk factor assessment interview was conducted for 100 out of the 109 FSWs who were reached through snowballing, to elicit data on their basic demographics, sexual activities and behaviours (including the age of sexual debut), reproductive history, menarche, sexually transmitted disease (STD) history, screening history, and smoking habit (past and present). Only FSWs of age 18 years and above and had been a sex worker for at least 6 months were included in the study. The Ethics and Protocol Review Committee of School of Biomedical and Allied Health Sciences, University of Ghana, approved this study (SBAHS/10161447/AA/MLS/2015-2016). Participants were fully informed about the purpose, procedures, risks, and benefits of participating in this study and Informed consent was obtained from all subjects.

### 2.2. Specimen Collection

Following the interviews, a Gynaecologist collected exfoliated cells from the cervix into tubes containing DNAgard™ (Biomatrica, San Diego, CA, USA) for HPV-DNA detection and genotyping. Samples were collected by single use, disposable equipment.

### 2.3. HPV Testing

HPV detection and typing were carried out by Nested multiplex PCR. [10]. A single consensus forward primer (GP-E6-3F) and two consensus back primers (GP-E7-5B and GP-E7-6B) were used for HPV DNA detection in the first round PCR. The PCR reaction mix of 25*μ*l contained 10X PCR buffer, 2.5mM MgCl_2_ 200*μ*M of each of the four deoxyribonucleoside triphosphates (dNTP's), 15pmols of each E6/E7 consensus primers, and 1.25 units of Taq polymerase enzyme. Five microliters (5*μ*l) of DNA extracts was used as a template for the amplification reactions using a thermal cycler (Robocycler Gradient 96, Strategene, USA). The cycling parameters for the first round PCR with E63F/E75B/E76B consensus primers were as follows: 94°C for four minutes, followed by 40 cycles of 94°C for one minute, 40°C for two minutes, 72°C for two minutes, and a single final elongation step of 72°C for 10 minutes. In the second round PCR, Primers for the identification of high-risk genotypes 16, 18, 31, 33, 35, 39, 45, 51, 52, 56, 58, 59, 66, and 68 and low-risk genotypes 6/11, 42, 43, and 44 were used. The primers were used in four cocktails, each containing four to five different primer pairs. Two microliters of first round PCR product, 15 pmols of forward, and reverse primers for genotyping were used. The other parameters remained the same as used in the first round PCR. However, the cycling parameters were as follows: 94°C for four minutes followed by 35 cycles of 94°C for 30 seconds, 56°C for 30 seconds, 72°C for 45 seconds, and a single final elongation step of 72°C for four minutes [10]. Positive and negative controls were included in each round of amplification.

The amplicons were resolved on 2% agarose gel stained with 0.5*μ*g/ml ethidium bromide. Ten microliters of each sample was added to 2*μ*l of orange G (10X) gel loading dye for the electrophoresis. Hundred base pair DNA molecular weight marker (Sigma, MO, USA) was run alongside the PCR products. The gel was prepared and electrophoresed in 1X TAE buffer using an electrophoresis tank at 80 volts for one hour and the gel photographed over an Ultraviolet (UV) transilluminator [10]. HPV genotypes were identified by comparing the molecular weight of the bands observed to positive control band and or the expected amplicon sizes in each primer cocktail.

### 2.4. Data Analysis

The data obtained through the questionnaire was checked for accuracy and entered into the computer using Microsoft Excel (2016) Programme and was analysed using SPSS version 20 (IBM Corp. Armonk, New York, USA). Exploratory analysis was first carried out to obtain descriptive statistics. Charts and tables were used to summarize data and display figures where appropriate. The number and proportion of HPV DNA positives and type-specific HPV infection were calculated. To assess the association between HPV DNA positivity and sociodemographic and sexual behavioural factors, odds ratios (ORs) and 95% CIs were calculated using logistic regression. In all statistical considerations a p-value <0.05 was considered statistically significance.

## 3. Results

### 3.1. Participant Characteristics

A total of 100 FSW, ages ranging from 18 to 45 years with a median age of 24 years, were interviewed. More than half (58%) of them were below 25 years. The level of education among the women was generally low, with the vast majority, 74% (74/100), having at most basic education while 6% (6/100) reported to be illiterates. The median length of sex work was 2 years with a range of 0.5-26 years. About a half (53%) of the sex workers reported engaging in other economic activities (mostly petty trading) while the rest (47%) were full time sex workers. Seventy-seven percent of the subjects who remember their age at sexual debut recalled it to be ≥16 years and 59% of them had at least a child. Condom use always was reported by 82% (82/100) of the study participants whereas oral contraceptive use was reported by 20%. Two (2%) subjects reported to be HIV positive while 14/100 (14%) had had gonorrhoea, 4/100 (4%) syphilis, 3/100 (3%) genital warts, and 2/100 (2%) herpes before. A third (33%) of them had ever smoked cigarette whist 17% are active smokers. Only 9/100 (9%) had ever undergone cervical cancer screening.

### 3.2. HPV Prevalence and Genotypes

**Figure 1:**
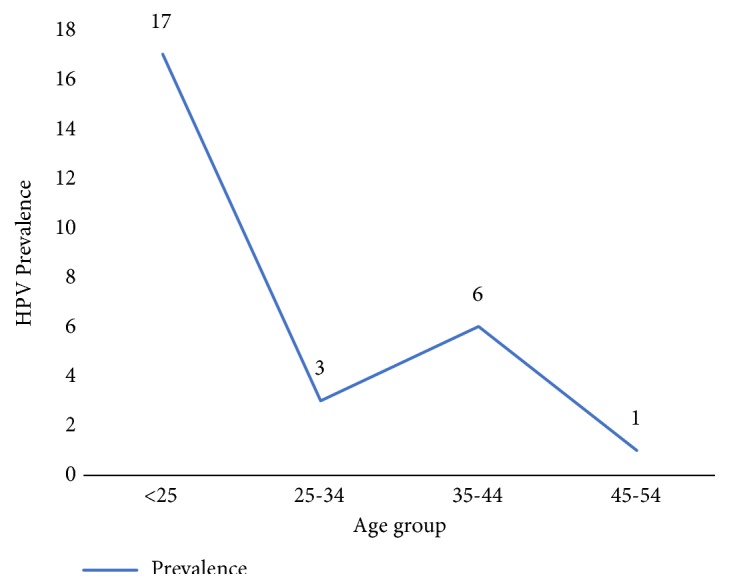
Age-specific HPV prevalence.

The overall HPV prevalence (any HPV) was 26% (26/100). Eleven genotypes were detected comprising 9 high-risk types and 2 low-risk types. The high-risk HPV types in order of decreasing prevalence were HPV-16 (8%; 8/100), HPV-35 (5%; 5/100), HPV-33/39/68 (3%; 3/100), and HPV-51/52/59 (2%; 2/100) HPV-18 (1%;1/100). HPV 42, (3%; 3/100) and HPV-43 (1%; 1/100) were the low-risk HPV types found. In addition 3% (3/100) had HPV that could not be typed by our method (Table 1). There were 21% single HPV infections (including the three that we could not type) and 5% multiple HPV infections. The 5 multiple infections comprised two each of quadruple- and double-infections and one triple infection. The highest HPV infection rate was among women aged below 25 years with 17% (17/100) (Figure 1). Of the HPV positive women, 73.1% (19/26) were infected with only high-risk types, 3.9% (1/26) were with only low-risk types, and 11.5% (3/26) had both high- and low-risk types.

Table 2 shows the cytological changes found in the high-risk HPV cases detected with 3 cases each of ASCUS, LSIL, and HSIL.

### 3.3. Risk Factors for HPV Infection

To identify putative risk factors for HPV infection, we performed univariate regression analysis and the results are presented in Table 3. HPV infection was found not to be significantly associated with, age, education, smoking, average number of clients per week, previous sexual disease, condom use, age at sexual debut, and parity. In contrast, the use of oral contraceptives was the only variable that significantly influenced HPV Infection (P=0.002). Female sex workers who used oral contraceptives had 19% reduced odds of having HPV infection compared to those who did not (OR=0.19, 95% CI 0.07-0.54).

## 4. Discussion

Studies have indicated that a high number of lifetime partners may lead to a higher transmission of HPV [6, 7]. The nature of work conducted by sex workers predisposes them to an increased risk of HPV infection. This study found a crude HPV prevalence of 26% among female sex workers in the Greater Region, which is higher than the WHO estimate of 19.5% HPV Prevalence in women from Western Africa at a given time [3]. This is consistent with existing reports of the elevated HPV prevalence in Female sex workers compared to women in the general population. Similar studies in other African countries have shown much higher prevalence: Madagascar (36.7) among 90 FSWs [11], Senegal (43.5%) among 681 FSWs [12], Tunisia (39.2%) among 51 FSWs [13], Burkina Faso (66.1%) among 360 FSWs [14], Kenya (55.6%); among 789 FSWs [15], South Africa (62.6%), among 99 FSWs [16]. These variations could be due to difference in sampling strategies and HPV assays employed [17] besides risk factors which are known to vary by region. Though we employed a comparatively more sensitive HPV assay (NMPCR) [10] to the conventional PCR (with either MY09-MY11 or GP5+-GP6+ primers) utilized in some of the studies above, we recorded a lower HPV prevalence compared to those studies. This may likely be due to the fact that 36% of the FSWs from the Burkina Faso study, 35.2 % from the Kenya study, and 50.3% from the South African study were HIV positive compared to the very low 2% in this study. Cervical HPV infections are substantially more common among women infected with HIV, compared with HIV-uninfected women with similar sexual histories due to their impaired immunity and this has been reported by several studies [18–20].

A high number of HPV positive FSWs (17/26), though not statistically significant, were below 25 years. This is consistent with reports in a global review of HPV prevalence among female sex workers [8]. This observation may be linked with acquisition of high rates of HPV following commencement of sex work. Secondly, this may be related to power play as older sex workers are better able to negotiate condom use than younger sex workers, although condom use does not provide full protection from HPV infection. This could also be ascribed to young sex workers enticing more clients than older sex workers culminating in an increased rate of exposure to HPV. It is however worth stating that more than half (58%) of our study subjects were within this age bracket and therefore this age trend could have also been due to this selection bias.

The high-risk HPV genotypes detected in decreasing order of prevalence were 16 (8%), 35 (5%), 33/39/68 (3%), 51/52/59 (2%), and 18 (1%). These genotypes are similar to what is seen in studies in Ghanaian women with and without cervical cancer and elsewhere in Africa but with varying individual genotype prevalence [21–25]. About 50% of high-risk-HPV genotypes detected in this study are covered by the Nona-valent vaccine (Gardasil® 9, Merck) [26] and therefore the introduction of this vaccine and ultimately a national vaccination policy would positively impact cervical prevention efforts in the country. However, the fact that not all high-risk-HPV detected in this and several other studies in Ghanaian women with and without cervical cancer are vaccine types means cervical screening will continue to play an important role in cervical cancer prevention efforts in the country even after the institution of a national vaccination policy. For example, HPV-35, the second most common HPV type found in this study, is not a vaccine type. In another study conducted in Ghana among HIV seropositive and negative women, HPV-35 was the commonest genotype detected and was significantly associated with Squamous intraepithelial lesions [25]. The low-risk-HPV genotypes detected, in decreasing order of prevalence, were 42 and 43. These two genotypes were the two common low-risk types detected in a study involving pregnant women attending antenatal clinic at the Korle-Bu teaching hospital in Accra [23].

Oral contraceptives use significantly influenced HPV infection (P=0.002), with about 19% decreased chance of HPV infection in oral contraceptive users compared to nonusers (OR=0.19, 95%CI 0.07-0.54). However, a systematic review of 19 epidemiological studies of the risk of genital HPV infection and oral contraceptive use concluded that there was no evidence for a strong positive or negative association between HPV positivity and ever use or long duration use of oral contraceptives [27]. There was lack of significant association of HPV infection with known risk factors such as smoking, age at sexual debut, number of sexual partners, and history of STI. These findings could be due to the small sample size and/or the reliability or otherwise of the information provided by the study subjects. The risk factors assessed in this study relied on self-reported data and therefore prone to both recall and social appeal bias.

This is the first report of cervical HPV and associated risk factors among FSW's in Ghana. Our study also has some limitations: this study was done on a small scale; therefore a larger study with a higher statistical power is needed to determine the extent of HPV infection in this population. Also, the convenience sampling method employed means the prevalence of HPV cannot be generalised to the whole female sex worker population in the Greater Accra.

## 5. Conclusion

This study found a high HPV prevalence among a cohort of female sex workers in the Greater Accra Region. A high number of the high-risk HPV seen in this population are vaccine preventable, providing additional compelling argument for implementing a national cervical cancer prevention plan including vaccination.

## Figures and Tables

**Table 1:** Type-specific HPV prevalence.

HPV type	Single infections	Multiple infections	Total (%)
			*n=100(100)*
***High-risk infections***			
16	4	4	8(8)
35	4	1	5(5)
33	2	1	3(3)
39	3	0	3(3)
68	1	2	3(3)
51	2	0	2(2)
52	1	1	2(2)
59	2	0	2(2)
18	0	1	1(1)
**sub-total**	**19**	**10**	**29**(29)
***Low-risk infections***			
42	2	1	3(3)
43	0	1	1(1)
**sub-total**	**2**	**2**	**4**(4)
**Un-typeable**			
X	3	0	3(3)
**Total (LR+HR+X)**	**24**	**12**	**36**(36)

**Table 2:** Cytologic changes in HR-positive individuals.

Cytology results	HR-Genotypes
NILM	16, 18, 68
NILM	16,35,68
NILM	33
NILM	39
HSIL	16,33,68
NILM	52
NILM	35
NILM	16
NILM	16
NILM	16
NILM	39
LSIL	16
LSIL	35
ASCUS	51
HSIL	51
NILM	35
HSIL	39
ASCUS	16,52
NILM	35
LSIL	33
NILM	59
ASCUS	59

**Table 3:** Results of univariate logistic regression analysis of risk factors for HPV infection.

Variables	HPV (+)	HPV (-)	OR (95% CI)	P-value
*Age(years)*				
<25	17	41	0.65 (0.26- 1.67)	0.38
25-34	3	23	3.46 (0.9412.67)	0.06
35-44	6	9	0.46 (0.15-1.46)	0.19
45-54	0	1	-	-

*Education*				
No formal education	1	5	1.81 (0.20-16.27)	0.60
Basic	18	56	1.38 (0.52-3.71)	0.52
Higher	7	13	0.59 (0.20-1.66)	0.31

*Time in prostitution (years)*				
<1	6	17	1.45(0.48-4.38)	0.51
1-2	6	27	1.47(0.55-3.94)	0.46
3-4	6	13	0.65(0.21-1.94)	0.44
≥5	8	15	0.58(0.20-.1.65)	0.31

*Full time sex worker*				
Yes	13	34	0.85 (0.35-2.08)	0.72

*Average clients per week*				
≤14	14	40	1.01 (0.41-2.71)	0.99
>14	12	34	1.05 (0.43-2.66)	0.92

*Contraceptive*				
Condom	20	62	1.55 (0.52-4.66)	0.44
Oral contraceptive	11	9	*0.19(0.07-0.54)*	*0.002∗*

*Age at sexual debut(yrs.)*				
<16	7	16	0.74 (0.27-2.09)	0.58
16-20	19	55	1.07 (0.38-2.93)	0.90
>20	0	3	-	0.99

*No. of Children*				
0	11	30	0.93 (0.37-2.30)	0.88
1-2	14	36	0.81 (0.33-1.99)	0.65
≥3	1	8	3.03 (0.36-25.47)	0.31

*Smoking History*				
Current smoking	7	10	0.42 (0.14-1.26)	0.12
Past smoking	9	24	0.91 (0.35-2.32)	0.84

*STI in the past*				
Yes	9	14	0.44(0.16-1.11)	0.11

*Past STI type*				
Genital warts	1	1	0.34(0.02-5.68)	0.46
Gonorrhoea	5	9	0.58(0.12-5.68)	0.36
Syphilis	2	2	0.33(0.04-2.50)	0.29
Herpes	1	1	0.34(0.02-5.68)	0.46
HIV	2	0	-	-

## Data Availability

The data used to support the findings of this study are included within the article.
